# Study on the destruction law of physical and shear properties of soil in mining disturbance

**DOI:** 10.1038/s41598-023-44943-5

**Published:** 2023-10-18

**Authors:** Qiya Qiao, Guangming Shi, Dejun Yang, Liyuan Wang, Xuan Zhang, Shichang Li, Xue-er Bai

**Affiliations:** 1 College of Geology and Mining Engineering, Xinjiang University, Ürümqi, 830017 China; 2 Beijing Geological Environment Monitoring Institute, Beijing, Beijing, 100097 China; 3 School of Environment Science and Spatial Informatics, China University of Mining and Technology, Xuzhou, 221116 China; 4 Xinjiang Baodi Engineering Construction Co. LTD, Ürümqi, 830046 Xinjiang China; 5 State Key Laboratory of Petroleum Resources and Prospecting, and Unconventional Petroleum Research Institute, China University of Petroleum, Beijing, Beijing, 102200 China

**Keywords:** Environmental sciences, Natural hazards

## Abstract

Underground mining activities can easily trigger surface subsidence and cause damage to surface soil. However, there is still a lack of studies on damaged soil, restricting ecological remediation in mining-induced subsidence regions to a certain degree. Focusing on the particular example of No. 4 Mine in Yili, Xinjiang, China, this study comprehensively combined field sampling, laboratory experiments, and data analysis to investigate the variation rules of basic physical properties and shear characteristics of soil samples. The latter had different subsidence degrees (0, 0–20, 20–40, and above 40 cm) and various depths (0– 10, 10–20, 20– 40, 40–60, and 60–80 cm). The experimental results show that: First, the natural density and dry unit weight of shallow soil in the serious-subsidence region were more significantly affected by mining-induced subsidence than the conditions in the deep layer, which also dropped with the increase in subsidence degree (with a mean drop rate of 7%). Second, serious subsidence could greatly counteract the positive effect of slight and moderate subsidence on the soil shear strength, with a drop rate of up to 30.7%. Third, compared with soil physical indices, mining-induced subsidence more easily affected shear strength indices. In particular, the soil samples taken from 0 to  10 cm depth in the slight subsidence area and 60–80 cm depth in the moderate subsidence area were most significantly affected by mining-induced subsidence, with PCA comprehensive scores of over 1.5. The present study can con-tribute to gaining in-depth knowledge of the damage characteristics of surface soil under mining-induced subsidence and provide a theoretical foundation for formulating reasonable coal mining strategies and ecological protection measures.

## Introduction

Coal mining plays a vital role in the global economy. For example, China’s coal production in 2021 exceeded 4.1 billion tons, with a year-on-year growth rate of 4.7%, occupying over half of the total global production^1,2^. Xinjiang is a famous coal base and ranks second in terms of coal resources among all provinces in China. Coal mining, on the one hand, has significantly promoted social and economic development in Xinjiang; however, on the other hand, surface collapse induced by underground coal mining also disturbs the physical, chemical, and biological properties of surface soil^3–7^, which can lead to significant changes in the soil-plant-underground water system, thereby causing damage to the local bioenvironment that has already been quite vulnerable. Therefore, gaining in-depth knowledge of the changes in the fundamental properties of surface soil under mining-induced collapse is essential. The present research results can provide new ideas for local governments in formulating ecological protection measures.

On account of increasing attention to the protection of the ecological environment, many scholars have conducted much research on the change in soil physical properties after mining-induced subsidence and achieved many results. Ahirwal et al.^8^ found that the pH value, conductivity, and bulk density increased significantly after mining activities, while the contents of soil nutrients (N, P, and K) dropped. Wang et al., focused on the coal deposits in the Jiahe Coal Mine, Xuzhou, and the Liuqiao Coal Mine, Huaihe, on a high-water-level plain; according to their results, for the soil samples at a depth of 0–20 cm in the deposit, bulk density increased gradually after the occurrence of subsidence. The bulk density of soil samples on the downslope or at the center of deposits increased most significantly^9,10^. By comparing soil physical and chemical properties in mining-induced subsidence and nonsubsidence areas, Zhao et al.^11^ reported that underground mining increased soil particle size and bulk density in the subsidence area. Zang et al., performed related tests and concluded that soil porosity with a subsidence age of 2 years was obviously higher than that in nonsubsidence regions. Soil porosity gradually recovered after subsidence for over three years^12^. Gu et al., carried out research in the Jiahe Mine administered by the Mining Bureau of Xuzhou and found that with the increase in subsidence degree, the soil’s total porosity and aeration porosity gradually dropped from the upslope of the subsidence basin to the bottom, accompanied by the enhanced compaction degree of soil^13,14^. In the aerated zone, the change in soil structure induced by mining subsidence affected water parameters, and the moisture contents in all the subsidence areas were lower than those in the nonsubsidence areas^15,16^. Based on previous literature results, despite a great deal of research on soil physical indices, most studies have focused on soil samples at the same depth with different subsidence degrees or at different depths with identical subsidence degrees while poorly combining the two factors (subsidence degree and depth) for overall analysis. Coal mining always induces a wide range of surface subsidence in actual production. Different regions varied obviously in subsidence degree, and the roots of different vegetation types were also distributed in the soil at different depths. Therefore, contrastive analysis simultaneously considering two factors is of greater practical significance.

Many scholars have also confirmed the tight correlation between soil shear strength and physical indices. Soil shear strength can reflect soil water-holding capacity and organic matter content to a certain degree. By combining all data on the relationship between critical shear stress and soil shear strength, Leonard and Richard investigated the meaning and universality of the relation between critical stress and shear strength in depth, reporting that saturated shear strength was the optimal soil property for predicting critical shear stress and run-off erosion^17^. Arvidsson and Keller^18^ revealed a strong correlation between soil moisture content and cohesive force; by taking soil in Sweden as an example, cohesive soil force dropped rapidly with increasing moisture content. Li et al.^19^ found that the slip capability of red soil on the Loess Plateau and yellow soil was negatively correlated with the sand content, cohesive force, water-stable aggregates, median diameter of aggregates, organic matter, and root density. At the Parana experimental station of the National Agricultural Special Technology Institute of Argentina, scholars concluded that soil shear strength was significantly correlated with bulk density and was sensitive to moisture content^20^. Hemmat et al., emphasized silty clay on an Isfahan University of Science and Technology farm in central Iran. They investigated the effects of organic manures (consisting of urban solid waste compost, air-dried sewage sludge, and farm manure of cows) on silty clay’s cohesive force and internal friction angle for seven years. It was concluded that for soil samples with different contents of organic matter or bulk densities, the variation in shear strength with moisture content could be uniform and explained by the consistency index^21^. However, scholars still need to investigate the influence of mining-induced subsidence on soil shear characteristics.

To sum up, many scholars have carried out very beneficial exploration on the influence of coal mining subsidence on soil characteristics, and have also achieved a lot of research results. Most of the relevant studies on coal mining subsidence are concentrated in the central plain area, the northwest wind–-sand area and the loess gully area, and the influence characteristics of soil properties are discussed based on typical geological conditions. The geological conditions of the low mountain and hilly area in western China are complex, with strong collapsibility and large surface fluctuation. The comprehensive impact evaluation of soil under the background of coal mining collapse in this area is rarely reported, and the following problems still need to be solved. First, although scholars have conducted much research on the change in soil physical properties under mining-induced subsidence, the studies mainly focused on single-scale variation in space while poorly investigating multiscale variation rules of soil characteristics. Second, most scholars selected a single soil index regarding physical and chemical properties, enzymatic activity, and biocenosis in mining-induced subsidence areas, while ignoring soil shear characteristics. Finally, which type of soil indices are most significantly subjected to mining-induced subsidence still needs to be determined^22–32^.

Therefore, this study focused on the surface subsidence area in the No. 4 Mine, Yili, Xinjiang, and performed field investigations, laboratory experiments, and data analysis to compare and analyze both physical indices (natural density, dry unit weight, and natural moisture content) and shear strength indices of different soil samples with different subsidence degrees and at different depths. The main components were extracted using principal component analysis (PCA), and comprehensive scores were calculated. The results show that the natural densities and dry unit weights of soil samples in the shallow layer of deep subsidence were more significantly affected under mining-induced subsidence than the values of the soil samples in the deep layer, and shear indices were more easily affected by mining-induced subsidence than physical properties. Furthermore, soil samples at a depth of 0–10 cm in the slight subsidence area and at a depth of 60–80 cm in the moderate subsidence area were most significantly affected by mining-induced subsidence, with comprehensive scores of over 1.5. The research feature of this paper is that it can clarify the spatial change characteristics of physical and mechanical properties of soil in Yili No. 4 mine subsidence area under the influence of coal mining collapse at different collapse degrees and depths, and evaluate the main influencing factors and corresponding influencing degrees of soil property indexes from two perspectives of basic test and mathematical statistics. The present research results can provide a theoretical foundation for formulating reasonable mining strategies and ecological protection measures, which is of great practical significance to protecting the ecological environment in Xinjiang coal mining areas and promoting green and sustainable development in the coal industry.

## Methods

**Figure 1:**
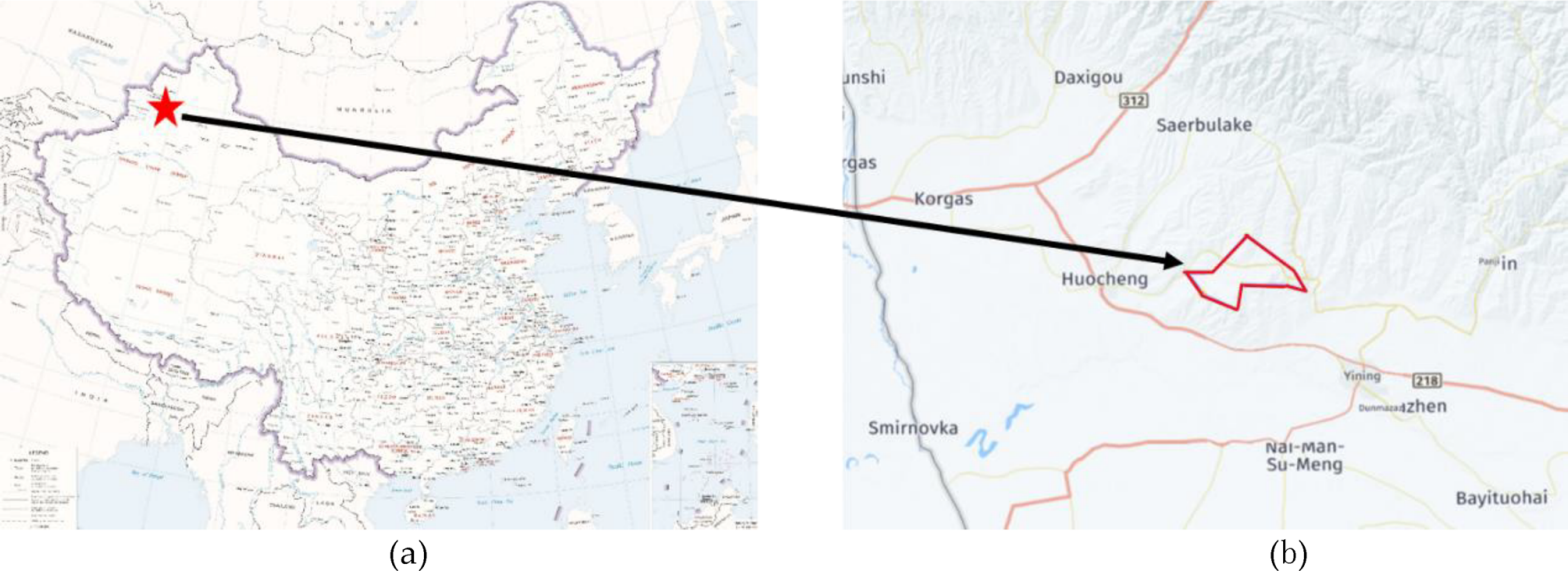
Illustration of the position of the research region. (**a**) A map of China, in which a red star marks the position of the present mine; (**b**) the position of the mine, in which a red block encloses the mine region; (download Link: Standard map site-http://bzdt.ch.mnr.gov.cn/download.html?superclassName=%25E4%25B8%25AD%25E5%259B%25BD%25E5%2585%25A8%25E5%259B%25BE%25EF%25BC%2588%25E8%258B%25B1%25EF%25BC%2589. With using the Microsoft PowerPoint to modify the map).

This study focused on the No. 4 coal mine owned in the research region, which is located in the southeastern part of Huocheng County, Kazakh Autonomous Prefecture, Yili City, Xinjiang Uygur Autonomous Region of northwestern China (see Fig. 1). The well has been developed for a long time with longwall retreating mining on strike. Many goafs have already been formed underground. The north area of the mining area is divided into 19 Wells (ore) fields, and the total planned production and construction scale of the mining area is 74.10 Mt/a. The coal seam in the mining area is shallow and the mining conditions are simple, which provides the conditions for small coal mining. Yili No. 4 well is located in the central north of the mining area, with a planned field size of 6.0 Mt/a. According to field investigation results, the subsidence area was 2400 m in length from east to west and 2300 mm in width from north to south, with an area of 4.72 km^2^. Step-pattern subsidence developed along the advancing direction, with a step drop of 30–60 cm.

The research region has a continental arid and semiarid climate, with a precipitation range of 186.30–387.70 mm and a mean value of 290.40 mm. The annual evaporation capacity is eight times greater than the annual precipitation. The mean maximum air temperature in July and August is 33.5 ℃, and the climate is lowest in January, with a monthly lowest value of − 21.3 ℃ and a mean value of 9.4 ℃. The mine is located in the low mountains and hills on the west side of Jieliangzi Valley at the border between Yining City and Huocheng County. The surface is always covered by herbaceous plants, with open sight, showing primitive hilly morphology. Many rough roads and crossing trenches can be observed. The terrain was intensively cut. Overall, the region is higher on the east side and lower on the west side, showing low hilly landforms. Paleozoic Permian bases were deposited, while Carboniferous and Devonian formations were distributed in the surrounding mountains. The outcropped strata include the Carboniferous, Triassic and Jurassic, Paleogene, Neogene, and Quaternary strata. The loess is 20–100 m in thickness. The shallow layer shows water collapsibility, with grass on the surface. The surface is mainly covered by mountainous calcareous soil and brown soil. The main constructive species is Seriphidium transiliense, mixed with many herbs, including Gramineae, Chenopodiaceae, Leguminosae, and Cruciferae.

### Soil sampling and laboratory measurements

#### Sampling scheme

As described above, the current subsidence area shows a step pattern with a step drop of approximately 30–60 cm. The mean and maximum subsidence depths are 3.55 and 4.25 m, respectively. More significant subsidence can be observed closer to the mining face. By reference to the method proposed by Zhou et al.^33^, this study divided the subsidence degrees into four types: nonsubsidence area, slight-subsidence area, moderate-subsidence area, and heavy-subsidence area, as listed in Table 1.

**Table 1:** Division of the surface soil in the mining-induced subsidence region with different collapse degrees^33^.

Collapse degree	The non-subsidence area	The slight-subsidence area	The moderate-subsidence area	The heavy-subsidence area
Staggered height/cm	0	0–20	20–40	> 40

**Figure 2:**
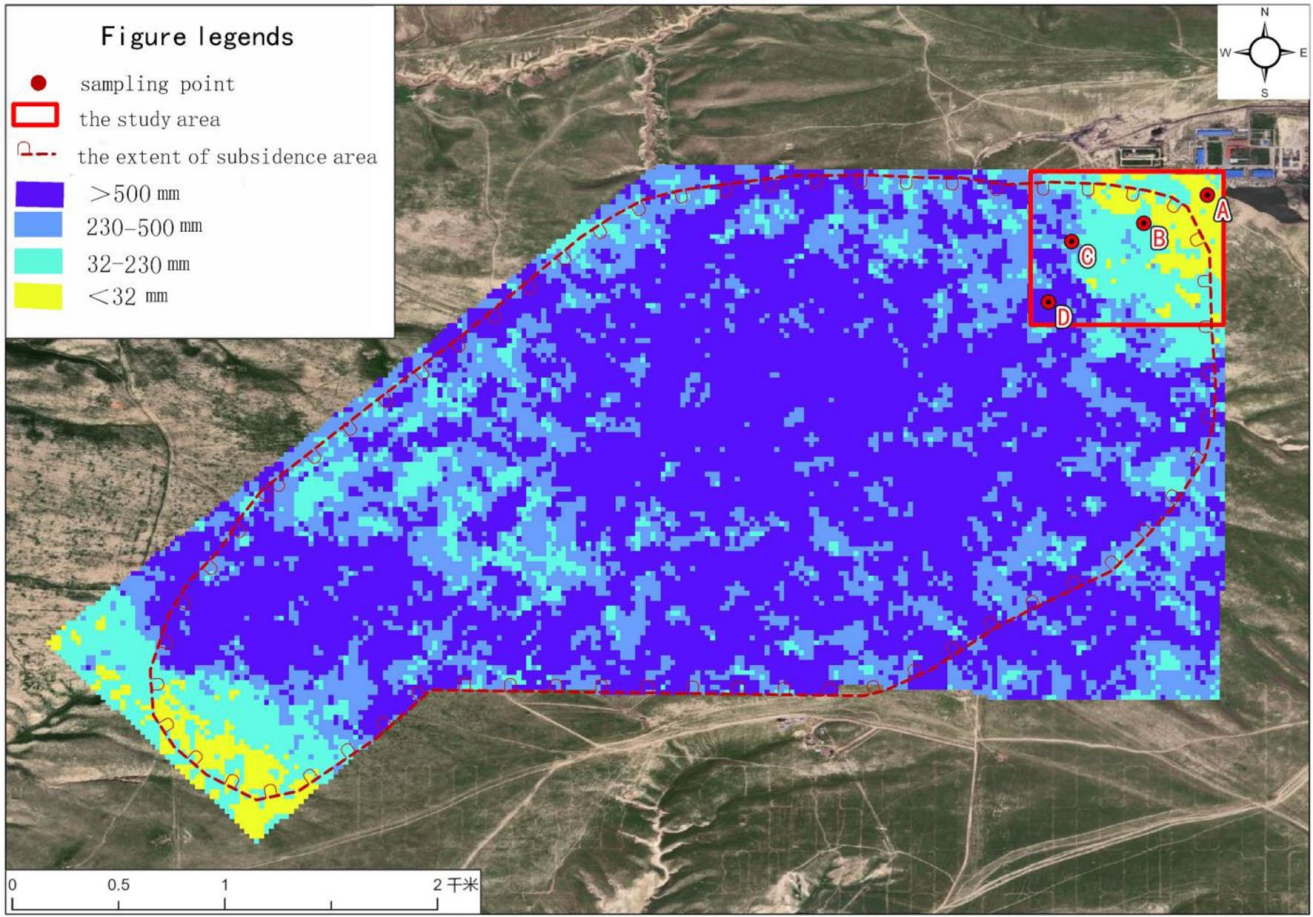
Comparison of D-inSAR surface deformation monitoring results and subsidence area calculated by section method. (The red box is the study area, A: the non-subsidence area; B: the slight-subsidence area; C: the moderate-subsidence area; D: the heavy-subsidence area) (download Link: Geospatial data cloud-https://www.gscloud.cn/sources/. With using the ArcGIS 10.3 to modify the map).

According to the Verification Report of Coal Resources Reserves in the No. 4 Well Field in the North Region of Yining Mining Area of Xinjiang and the production geological report of the original mine, the current mining coal seam is 21-1 coal seam in the 11 mining area, the coal seam inclination is 6°–8°, and the uniaxial compressive strength under the saturated state of the roof is between 0.2 and 15 MPa, which is a weak rock. In this analysis, the caving zone and water-conducting fracture zone method were used to calculate the caving zone and water-conducting fracture zone of each coal seam according to the soft (10–20 MPa) overburden lithology formula recommended in the Code for Hydrogeological Engineering Geological Exploration of Mining Area (GB/T 12719-2021). According to the Verification Report of Coal Resources Reserves in the No. 4 Well Field in the North Region of Yining Mining Area of Xinjiang and the production geological report of the original mine, the current mining coal seam is 21-1 coal seam in the 11 mining area, the coal seam inclination is 6°–8°, and the uniaxial compressive strength under the saturated state of the roof is between 0.2 and 15 MPa, which is a weak rock. In this analysis, the caving zone and water-conducting fracture zone method were used to calculate the caving zone and water-conducting fracture zone of each coal seam according to the soft (10–20 MPa) overburden lithology formula recommended in the Code for Hydrogeological Engineering Geological Exploration of Mining Area (GB/T 12719-2021). Moreover, based on synthetic aperture radar technology D-inSAR technology, considering that mining in the mine will start in 2018, it is found that collapse will begin in 2020 on the remote sensing map, so the data of Sentinel-1 in January 2020 and Sentinel-1 in January 2023 are obtained to complete the ground subsidence deformation monitoring of the mine area of Yili No. 4, and the remote sensing satellite map is superplaced. It can be found that the subsidence range calculated by the profile method is consistent with the surface subsidence range processed by D-inSAR, so the correctness of the location selection of sampling points in the non-subsidence area can be determined, and the locations of sampling points in the other three areas can be selected based on the monitoring data and the scattered height of the cracks in the sampling area. The monitoring results of D-inSAR surface deformation and the subsidence range calculated by section method are shown in Fig. 2. Moreover, based on synthetic aperture radar technology D-inSAR technology, considering that mining in the mine will start in 2018, it is found that collapse will begin in 2020 on the remote sensing map, so the data of Sentinel-1 in January 2020 and Sentinel-1 in January 2023 are obtained to complete the ground subsidence deformation monitoring of the mine area of Yili No. 4, and the remote sensing satellite map is superplaced. It can be found that the subsidence range calculated by the profile method is consistent with the surface subsidence range processed by D-inSAR, so the correctness of the location selection of sampling points in the non-subsidence area can be determined, and the locations of sampling points in the other three areas can be selected based on the monitoring data and the scattered height of the cracks in the sampling area. The monitoring results of D-inSAR surface deformation and the subsidence range calculated by section method are shown in Fig. 2.

During field sampling, soil samples were collected from the stably settled region. Field operation was performed to ensure the safety of sampling workers. At the sampling points, the soil profile with a depth of 1 m was dug with a shovel. Considering that the collapse crack develops downward in the shape of an inverted triangle, the width becomes larger the closer it is to the surface, and the width becomes smaller the deeper it develops, 20 cm is further subdivided in the upper layer, and five depths are finally selected. From top to bottom, the soil samples were collected at five depths (0–10, 10–20, 20–40, 40–60, and 60–80 cm). In each layer, four small cutting-ring and three large cutting-ring soil samples were collected with the soil cutter and taken to the laboratory for measurements. Figure 2 displays the sampling process. The sampling scheme is shown in Table 2.

**Table 2:** Sampling scheme.

Species	Working method	Job content	Quantity
Sampling	Field sampling	Different size undisturbed ring cutter sample	80 undisturbed small ring knife samples 60 original large ring knife samples
Physical property index test	Ring tool method	Natural density, dry weight	60 original large ring knife samples
	Drying method	Moisture content	
Mechanical property index test	Direct shear apparatus	Shear strength, cohesion, angle of internal friction	A total of 80 groups of undisturbed small ring cutter samples in 20 points
Data processing and analysis	Mathematical statistics and probability theory	Index change characteristics	–

#### Basic physical test

According to the specifications in the Standard for Geotechnical Testing Methods (GB/T 50123-2019)^34^, the natural densities, dry unit weights, and natural moisture contents of the 60 collected samples were measured.

During the present tests, the surplus oil on the two sides was removed and flattened with a ring cutter (φ79.8 × 20 mm). After the external wall of the ring cutter was cleaned, the weights of the ring cutter and soil samples were taken and recorded with an accuracy of up to 0.1 g. Next, the ring cutter was placed in the 101-type electric-heating air-blast drying oven and dried at 105 ℃ for 8 h. The weights after cooling were also taken. In this study, the volume of the ring cutter was 100 cm^3^. Finally, the natural density, dry unit weight, and natural density can be calculated according to Eqs. (1)–(3):

1 $$\rho = \frac{{m_{0} }}{V}$$

2 $$\rho_{d} = \frac{{m_{d} }}{V}$$

3 $$\omega = \left( {\frac{{m_{0} }}{{m_{d} }} - 1} \right) \times 100$$

where $\rho$ is the natural density, g/cm^3^; $m_{0}$ is the dry unit weight, g/cm^3^; $V$ is the natural soil weight, g; $\rho_{d}$ is the weight of dried soil, with a unit of g; $m_{d}$ is the volume of cutter ring cutter, cm^3^; and $\omega$ is the moisture content, %.

#### Direct shear test

To obtain the internal friction angle and cohesive force of the soil, a direct shear test was performed on 80 samples according to the Standard for Geotechnical Testing Methods (GB/T 50123-2019)^34^. Considering that the soil structure can become looser under mining-induced subsidence, consolidation may counteract the effect. Accordingly, a quick shear test was performed.

The detailed test process is described below. According to the specifications and soil hardness degrees, considering the overburden pressure on the undisturbed soil under natural conditions, the vertical pressure was uniformly set as 50, 100, 150, and 200 kPa. The fix stopper was inserted by aligning both the upper and lower boxes. The watertight plate was set in the lower box, and the ring cutter with the sample was flat down. By aligning the mouth of the shear cell, the watertight plate was set on the top of the sample. The sample was slowly pushed into the shear box, and the ring cutter was removed. The steel column in the front end of the upper box was adjusted to be in exact contact with the dynamometer. After resetting, the pressure plate and frame-work were added in succession, and the initial reading was recorded. After the application of vertical pressure, the fixed stopper was pulled out. The stopwatch was started, and shear was applied at a 0.8 mm/min rate. The readings were periodically recorded until shear failure. Finally, the test results were calculated as follows:

4 $$\tau = CR*10/A$$

where τ is the shear stress force(kPa); C is the calibration coefficient of the dynamometer(N/0.01 mm); R is the reading of the dynamometer(0.01 mm); and A is the initial sample area(cm^2^).

### Principal component analysis (PCA)

Classical PCA is an essential branch of multivariate statistical analysis. The basic idea is to reduce many original indices to a few mutually independent comprehensive indices^35^. PCA holds the opinion that there necessarily exist common factors that play a dominant role among many relevant factors. Accordingly, by analyzing the structural relations in the correlation matrix of the original variables, several comprehensive indices affecting economic processes can be sought, which can not only retain original information but also realize irrelevancy, thereby showing superiority to the original variables. Through PCA, scholars can quickly grasp principal contradictions in solving complex problems^36,37^. Using a few new variables to replace many original variables can avoid information overlap and simultaneously achieve the goal of di-mensionality reduction^38^ to ensure both the accuracy and truth of the research results. Scholars have extensively applied PCA in many domains and gained favorable results.

The detailed calculation procedure is described below.

- Standardize the original data to eliminate the effect of dimension

Assuming original m target variables for PCA $x_{1} ,x_{2} , \ldots x_{m}$ and n evaluation objects, $x_{ij}$ denotes the value of the j-th index of the i-th evaluation object. Accordingly, various indices $x_{ij}$ can be converted to standardized $\tilde{x}_{ij}$ indices.

5 $$\left\{ {\begin{array}{*{20}l} {\tilde{x}_{ij} = \frac{{x_{ij} - \overline{x}_{j} }}{{s_{j} }},(i = 1,2, \ldots ,n;j = 1,2, \ldots ,m)} \\ {\overline{x}_{j} = \frac{1}{n}\sum\limits_{i = 1}^{n} {x_{ij} } } \\ {s_{j} = \frac{1}{n - 1}\sum\limits_{i = 1}^{n} {(x_{ij} } - \overline{x}_{j} )^{2} ,(j = 1,2, \ldots ,m)} \\ \end{array} } \right.$$

where $\overline{x}_{j}$ and $s_{j}$ denote the sample mean and standard deviation of the j-th index. Correspondingly, $\tilde{x}_{i} = \frac{{x_{i} - \overline{x}_{i} }}{{s_{i} }},(i = 1,2, \ldots ,m)$ can be referred to as the standardized index variable.

- Establish the correlation matrix R among variables

The correlation coefficient matrix can be written as:

6 $$\left\{ {\begin{array}{*{20}l} {R = (r_{ij} )_{m * m} } \\ {r_{ij} = \frac{{\sum\limits_{k = 1}^{n} {\tilde{x}_{ki} \cdot \tilde{x}_{kj} } }}{n - 1},(i,j = 1,2, \ldots {\text{,m)}}} \\ \end{array} } \right.$$

where $r_{ii} = 1$, $r_{ij} = r_{ji} ,r_{ij}$ denotes the correlation coefficient between the i-th and the j-th indices ( ).

- Calculate the eigenvalue of the correlation coefficient matrix R, denoted as $\lambda_{1} \ge \lambda_{2} \ge \cdots \ge \lambda_{m} \ge 0$, and the corresponding eigenvectors $u_{1} ,u_{2} , \ldots ,u_{m} ,$ in which $u_{j} = (u_{1j} ,u_{2j} , \ldots ,u_{nj} )^{T}$ constituting the following m new index variables from eigenvectors:
  7 $$\left\{ {\begin{array}{*{20}l} {y_{1} = u_{11} \tilde{x}_{1} + u_{21} \tilde{x}_{2} + \cdots + u_{n1} \tilde{x}_{n} } \\ {y_{2} = u_{12} \tilde{x}_{1} + u_{22} \tilde{x}_{2} + {{\cdot\cdot\cdot}} + u_{n2} \tilde{x}_{n} } \\ { \ldots \ldots \ldots \ldots \ldots \ldots \ldots \ldots \ldots \ldots \ldots \ldots } \\ {y_{m} = u_{1m} \tilde{x}_{1} + u_{2m} \tilde{x}_{2} + {{\cdot\cdot\cdot}} + u_{nm} \tilde{x}_{n} } \\ \end{array} } \right.$$
  where $y_{1}$, $y_{2}$, and $y_{m}$ denote the first, second, and m-th principal components (PCs), respectively.
- Obtain principal components and calculate overall scores

Calculate the contribution ratio of the eigenvalue $\lambda_{j} (j = 1,2, \ldots ,m)$ and the cumulative contribution ratios:

8 $$b_{j} = \frac{{\lambda_{j} }}{{\sum\limits_{k = 1}^{m} {\lambda_{k} } }}(j = 1,2, \ldots {\text{,m)}}$$

9 $$\alpha_{p} = \frac{{\sum\limits_{k = 1}^{p} {\lambda_{k} } }}{{\sum\limits_{k = 1}^{m} {\lambda_{k} } }}$$

where $y_{j}$ is the information contribution ratio of the principal component , $y_{1} ,y_{2} , \ldots {\text{,y}}_{p}$ denotes the cumulative contribution ratio of the principal components. When $\alpha_{p}$ is close to 1 (for example, $\alpha_{p} = 0.85,0.90,0.95$), the first $p$ index variables $y_{1} ,y_{2} , \ldots {\text{,y}}_{p}$ can be selected as $p$ principal components to replace the original $m$ index variables for comprehensive analysis.

Calculate the comprehensive scores

The comprehensive score can be calculated as follows:

10 $$Z = \sum\limits_{j = 1}^{p} {b_{j} y_{j} }$$

where bj is the information contribution ratio of the j-th principal component.

## Results

### Analysis of the measured basic physical properties

#### Natural density and dry unit weight

**Figure 3:**
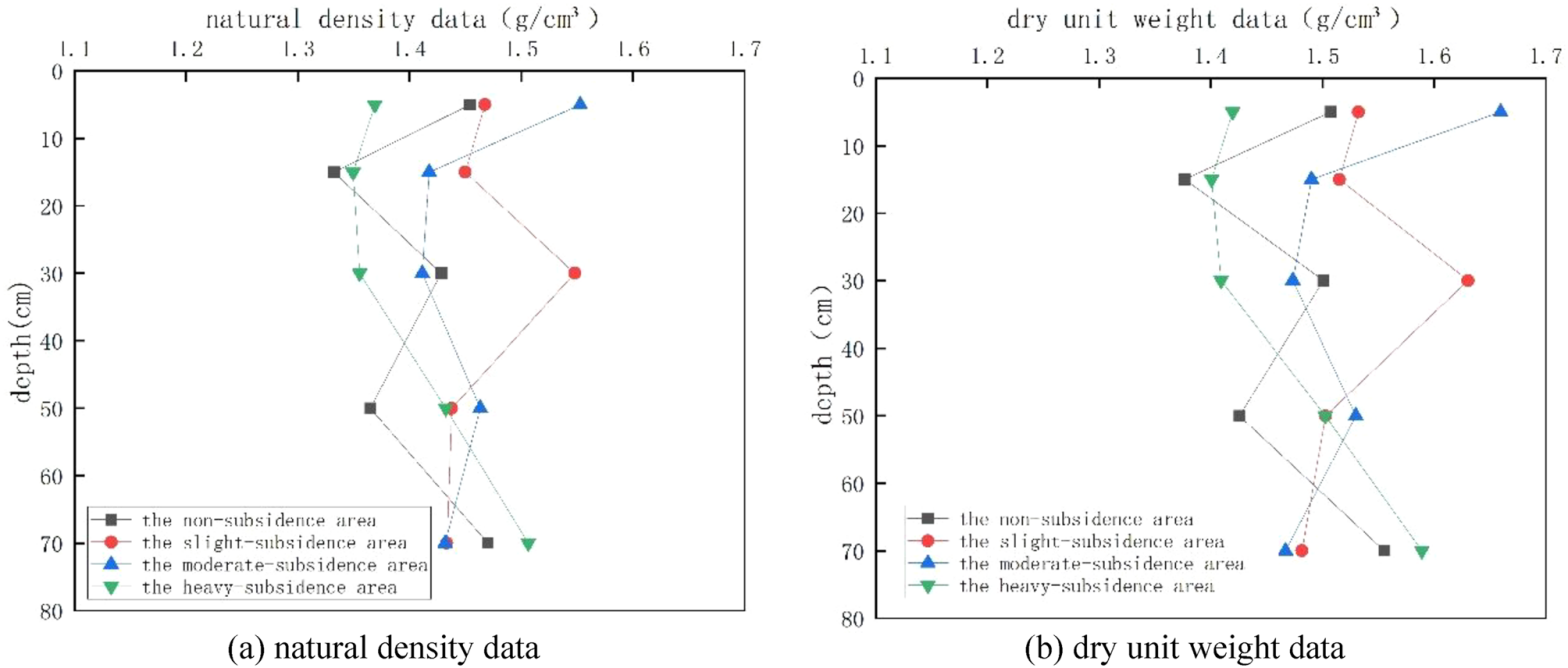
Variation tendency of natural density and dry weight with the subsidence degree.

In the test, three sets of parallel tests were designed for each of the 20 points, and a total of 60 sets of natural density data and 60 sets of dry weight data were obtained. After data processing, index change curves were drawn, as shown in Figure 3.

When the depth of the lower layer is less than 20 cm, the soil layer in the four regions is above the surface, and external disturbance and external environment erosion will cause the soil layer to loose, which becomes obvious with the increase of the depth. As the depth increases, the soil density decreases. Therefore, in the range of 0–20 cm, due to the influence of the above factors, the soil layer becomes loose, the pores between soil particles become larger, and the natural density and dry weight decrease. For the abrupt change point of 20–40 cm layer, the reason is that as the depth increases gradually, when it is greater than 20 cm, the soil layer at this time is located below the surface, resulting in the soil being less disturbed by external forces and weaker by environmental erosion. The bonding effect between soil skeleton and soil particles is sufficient to support the soil structure to resist external disturbances and erosion. Therefore, with the increase of depth, its natural density and dry weight show a larger law, and with the increase of collapse degree, the soil skeleton is more solid, so the change trend is not as obvious as that in unsubsidence area and light subsidence area. When the depth continues to increase, the soil layer in the uncollapsed area and the mildly collapsed area reaches the 40–60 cm horizon, at this time, the soil disturbance increases significantly. The soil skeleton in these two areas is not enough to support the disturbance of external forces, the soil is destroyed, the soil skeleton is loose, and the density of the soil is reduced, while the soil layer in the severely collapsed area and the moderately collapsed area is thicker than that in the mildly collapsed area and the non-collapsed area. The soil skeleton becomes denser and the interaction between soil particles is enhanced, so it can resist external disturbance, so the soil density increases. When the depth continues to increase to 60–80 cm, the soil density in the heavy subsidence area is the largest among the four areas, because it has the deepest depth, more dense soil particles and the smallest pores, so the natural density and dry weight of the soil are the largest.

#### Moisture content

**Figure 4:**
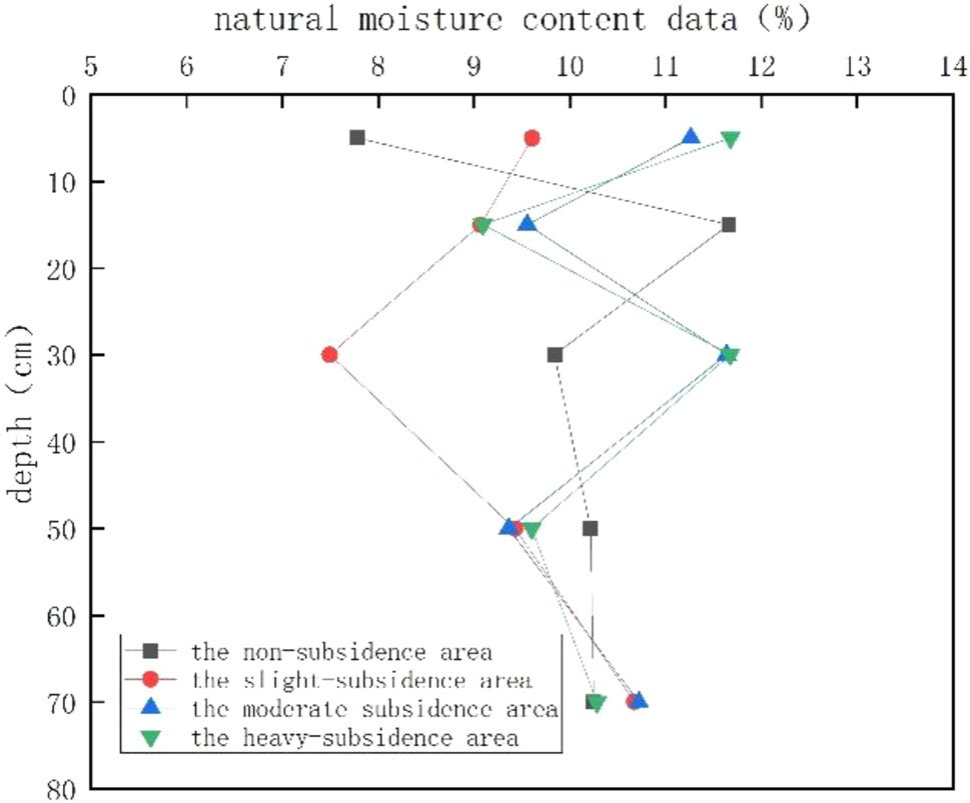
Variation tendency of natural moisture content with the subsidence degree.

In the test, three sets of parallel tests were designed for each of the 20 points, and a total of 60 sets of moisture content data were obtained. After averaging the results, soil moisture content of 20 sampling points was obtained. After data processing, the index change curve was drawn, as shown in Fig. 4.

The subsidence area is affected by surface runoff and rainfall, resulting in the increase of moisture content in the soil mass. This phenomenon increases with the increase of the collapse degree. Therefore, the natural moisture content of the soil mass in the unsubsidence area increases with the increase of the collapse degree between 0 and 10 cm. When the depth reaches 10–20 cm, the soil layer in the non-subsidence area is above the surface. As the soil is loose, the natural density decreases and the void increases. However, as the depth continues to increase, the soil layer becomes dense, making the residual surface runoff water trapped in the dense soil layer structure and unable to continue to penetrate, resulting in an abnormal increase in its natural water content. As for subsidence area, due to soil disturbance, the soil is loose and the pores increase, and the water in the soil is affected by gravity and flows downstream, resulting in the decrease of its natural water content. When the depth continues to increase, the unsubsidence area and the mild subsidence area become weaker due to the disturbance and environmental erosion, and the soil skeleton is intact, so the water content decreases. The water content of moderate subsidence area and severe subsidence area increases due to the influence of surface runoff in unsubsidence area and light subsidence area. When the depth increases to more than 10–20 cm, the external disturbance in the unsubsidence area and the mild subsidence area is enhanced, the soil structure is destroyed, and the water storage capacity is enhanced, so the water content increases again. For the moderate subsidence area and the severe subsidence area, there is a special point at 20–40 cm, which can be understood as the difference in permeability. It has capillary blocking effect at the interface, and has a certain water storage function, which leads to the increase of water content. As the depth increases, the soil structure becomes more dense and the pores decrease, so the water content decreases. When the depth reaches 60–80 cm, the soil structure is stable enough, so the natural water content is similar for the four regions.

### Analysis of direct shear test results

#### The shear strength

**Figure 5:**
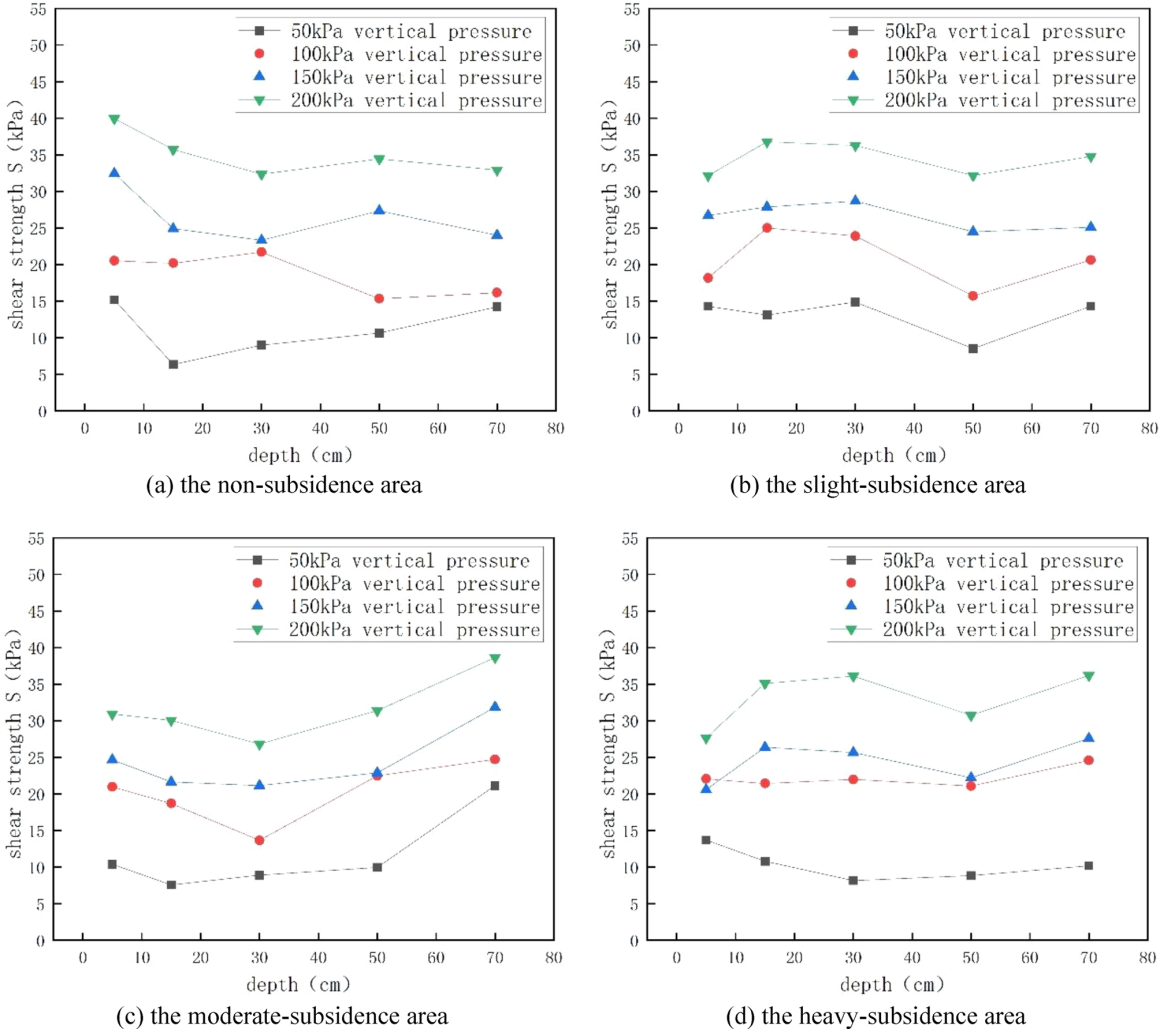
Shear strength data of different soil samples with different subsidence degrees and under different overburden pressure.

A direct shear test was performed on field-collected soil samples. The shear strength was calculated via the earlier presented Eq. (4). Combining the calculated shear stress and the displacements calculated by the periodic readings in the test, the correlation curve between shear stress and shear displacement can be plotted. The peak or stable value in the curve was selected as the shear strength S, as shown in Fig. 5. Example text under a subsection. Bulleted lists may be used where appropriate, e.g. cohesive force and internal friction angle

**Figure 6:**
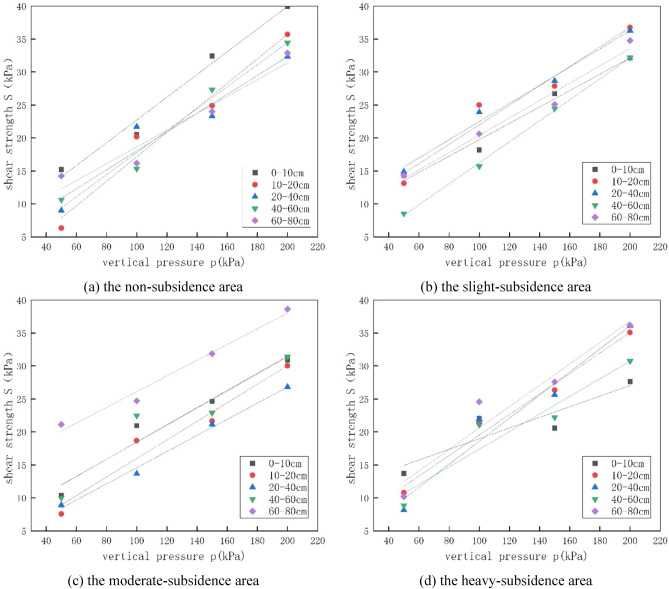
Curve of the relationship between shear strength and vertical pressure with different subsidence degrees and under different overburden pressure.

After the shear strength was obtained, the correlation between the shear strength and the applied vertical pressure was plotted. Using the Origin 8.0 data analysis and plotting software, the linear relationship was fitted to derive the relation between the cohesive force c and the internal friction angle φ, as shown in Fig. 6.

The correlation curve between S and P can be plotted by using the shear strength S and the unit vertical pressure P as the y- and x-axes, respectively. The soil shear strength, internal friction, and cohesive force were determined according to Coulomb’s law. The inclination angle denotes the soil’s internal friction angle φ, while the intercept of the line on the y-axis denotes the soil’s cohesive force C. Accordingly, the shear strength indices of the soil samples under different subsidence degrees were calculated, the R-square value of the fitted line is obtained, which conforms to the molar Coulomb criterion as shown in Table 3, and the shear strength index of soil under different mining collapse degrees is obtained, as shown in Table 4.

**Table 3:** The shear strength and vertical pressure are fitted to the linear R-squared value.

Subsidence degrees	0–10 cm	10–20 cm	20–40 cm	40–60 cm	60–80 cm
The non-subsidence area	0.98094	0.96881	0.92607	0.97508	0.93869
The slight-subsidence area	0.98145	0.95244	0.98734	0.99868	0.97581
The moderate-subsidence area	0.95907	0.95717	0.99351	0.89559	0.98157
The heavy-subsidence area	0.8249	0.98248	0.95734	0.91597	0.93516

**Table 4:** Shear strength indices.

Sample depth	Subsidence degrees	Cohesive force c(kPa)	Internal friction angle φ(°)
0–10 cm	The non-subsidence area	5.48566	9.778857434
	The slight-subsidence area	7.30949	7.081008974
	The moderate-subsidence area	4.42343	8.107240036
	The heavy-subsidence area	11.90157	3.931185481
10-20 cm	The non-subsidence area	1.41121	10.51611603
	The slight-subsidence area	7.24792	8.392870032
	The moderate-subsidence area	1.90245	8.010628166
	The heavy-subsidence area	3.96865	8.843741445
20–40 cm	The non-subsidence area	3.69894	8.151037138
	The slight-subsidence area	8.70076	7.847632451
	The moderate-subsidence area	2.33537	6.978292522
	The heavy-subsidence area	1.11894	9.916794384
40–60 cm	The non-subsidence area	1.08057	9.471984792
	The slight-subsidence area	0.30215	9.052843048
	The moderate-subsidence area	5.52649	7.362396219
	The heavy-subsidence area	4.02179	7.607403185
60–80 cm	The non-subsidence area	8.37693	5.574980198
	The slight-subsidence area	9.25027	6.143096597
	The moderate-subsidence area	15.18627	6.118173984
	The heavy-subsidence area	3.37975	9.873423426

**Figure 7:**
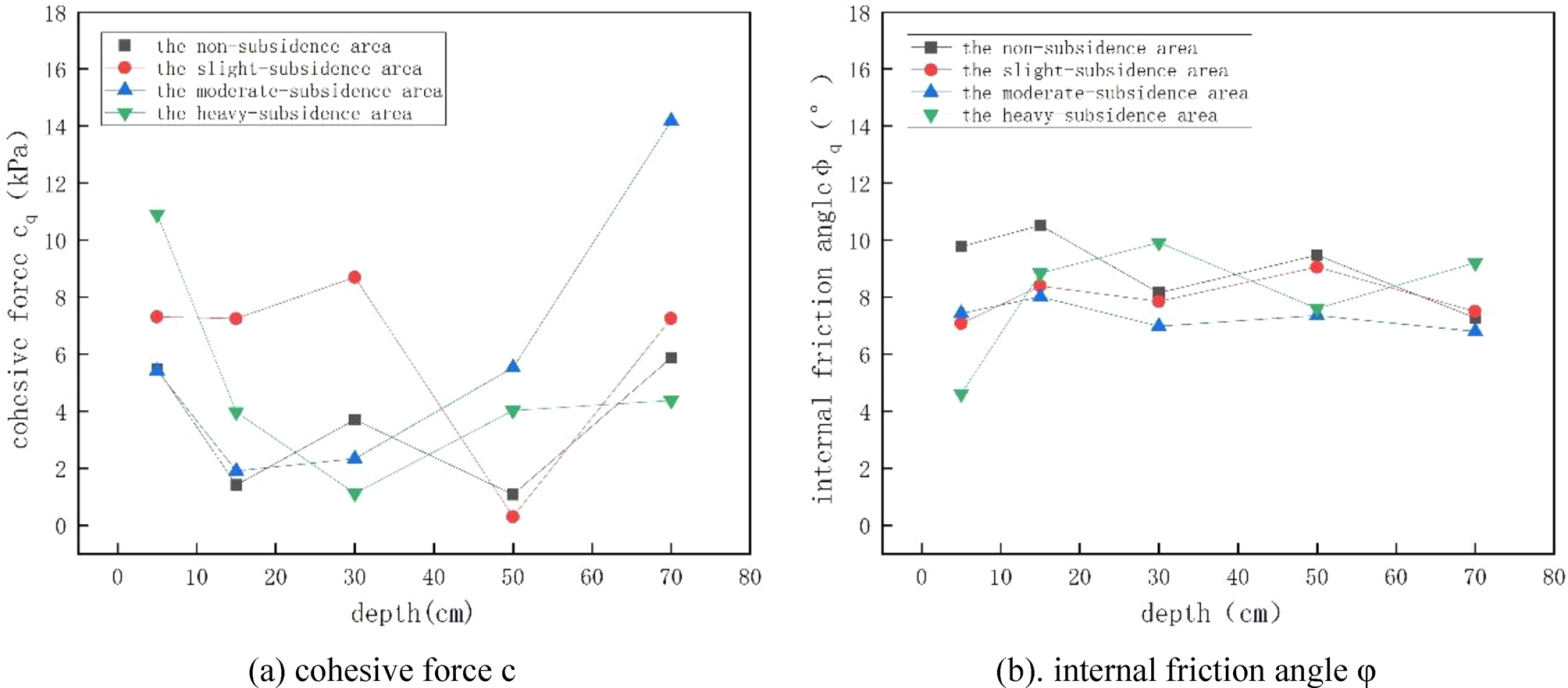
Variation trend of cohesion and internal friction Angle under different depth and collapse degree.

The obtained shear strength index was processed to draw a graph, and the changing trend of cohesion and internal friction Angle under different depths and different collapse degrees was analyzed, as shown in Fig. 7.

**Figure 8:**
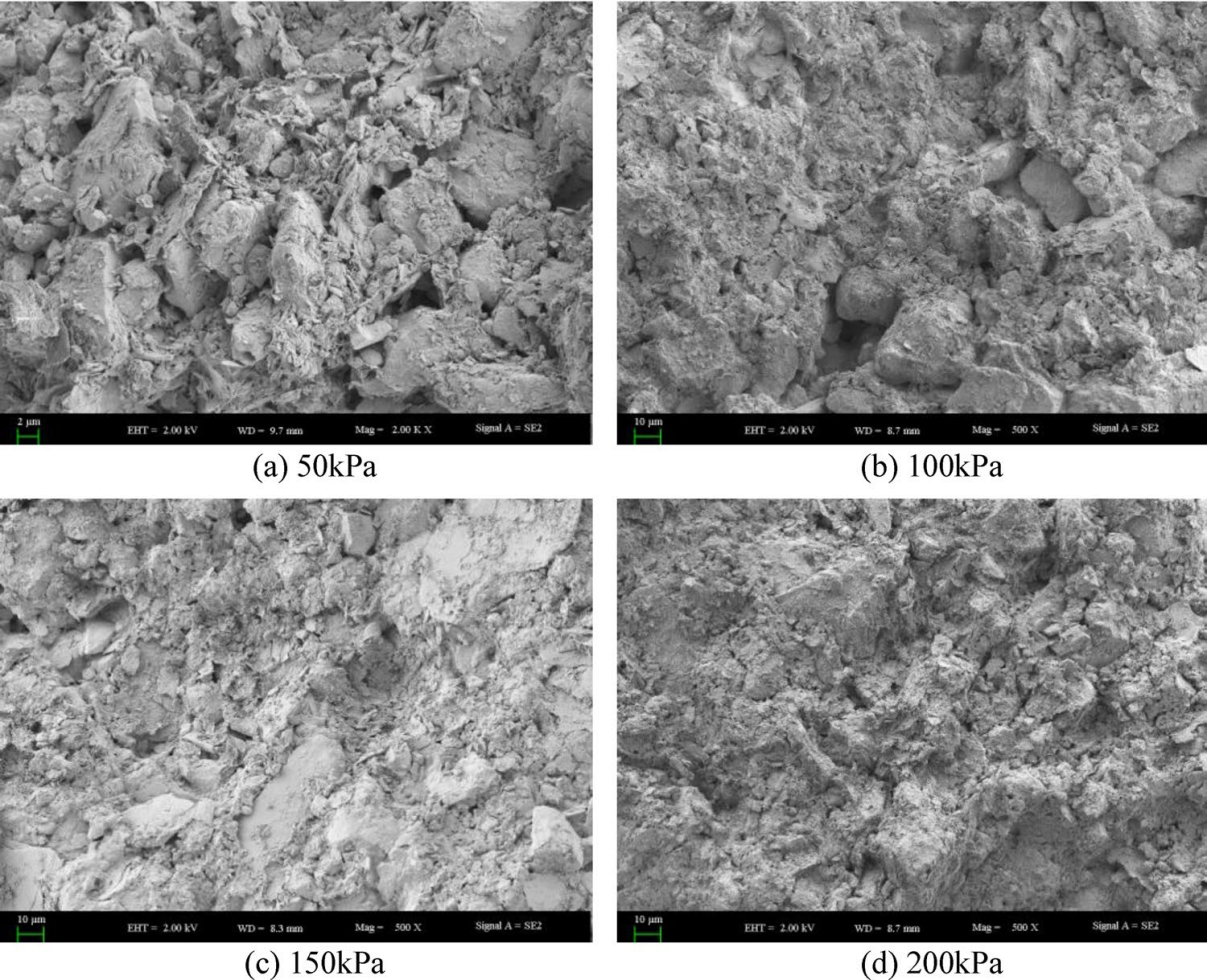
500-fold scanning electron microscope (SEM) images of soil at different overlying pressures at 40–60 cm depth in non-subsidence area.

For the 4 regions and 5 different depths, the shear strength increases with the increase of overlying pressure. This is because with the increase of overlying pressure, the pores in the soil are squeezed out, the soil structure is more dense, and the bonding between soil particles is enhanced, so the strength increases, irrespective of whether there is collapse or depth. The damaged sample was subjected to SEM scanning electron microscopy, and the results were shown in Fig. 8. The picture under 500 times is shown in the following figure. It can be seen from the figure that under the same magnification, with the increase of overlying pressure, the pores become smaller, the bonding between soil particles becomes stronger, the soil skeleton is filled with more soil particles, and the soil tends to be dense, so its shear strength gradually increases. The shear strength is affected by many factors such as soil disturbance, over-lying pressure and water content. However, with the increase of depth, when the depth is less than 20 cm, the shear strength of most soils decreases with the increase of depth. This is because the soil layer at this depth is in the air and subject to external disturbance and environmental erosion, and the soil layer is loose, especially when the soil layer tends to the boundary of 20 cm. At this time, the soil is affected by the step and the disturbance effect is obvious. Therefore, its shear strength is the lowest. The reason for the different laws in the mild subsidence area and the severe subsidence area is that the positive effect of overlying pressure on the shear strength is greater than the negative effect of coal mining and water content on the shear strength of soil samples, which improves the shear strength to some extent. However, when the depth continues to increase, the soil is completely inside and the degree of external influence decreases. The shear strength of the soil is determined by the depth of the soil and the disturbance caused by underground mining. For the mild subsidence area, the soil structure is not stable, and the disturbance effect generated by underground mining is greater than the influence of soil depth on the shear strength, resulting in a decline in the shear strength of some soils (the depth is less than 50cm). However, with the further increase of depth, the soil structure becomes denser. At this time, the influence of soil depth on the shear strength is gradually greater than the disturbance of underground mining, so the shear strength gradually increases. For the moderate subsidence area and the severe subsidence area, the influence of soil depth is obviously greater than the disturbance effect because the soil layer structure has reached stability, so the overall shear strength of soil shows a trend of increasing, and the overlying pressure has little influence on the shear strength. Most of the values of the internal friction Angle range from 7° to 10°. With the deepening of the collapse degree, the soil becomes denser and the cohesion and internal friction Angle gradually increase. However, the deep soil layer is disturbed with the development of mining, so the disturbance degree of the soil layer increases with the increase of the depth, resulting in the smallest cohesion of the soil sample in the deepest subsidence area. The soil layer disturbance in the area of severe subsidence is larger and destroys the soil structure, while the disturbance in the area of moderate, mild and unsubsidence is smaller and the soil layer structure is smaller. The reason that the surface internal friction Angle is the smallest in the heavy subsidence area is that the water content is the highest at this point, which will increase the cohesion between soil particles and reduce the size of the internal friction Angle.

### PCA results

This study collected soil samples at 20 points with four subsidence degrees at five different sampling depths. It analyzed the effects of mining-induced subsidence on soil samples and some main soil properties influenced by subsidence with SPSS2.0 (statistical analysis software). The original data were standardized to eliminate the adverse effects that may be induced in different dimensions. To validate whether the data were suitable for PCA, this study also performed the KMO test and Bartlett’s test of sphericity on the PCA data. The results are listed in Table 5. According to the results, the value of KMO was 0.502 (> 0.5), and the value of Sig was smaller than 0.05, suggesting suita-bility for PCA.

**Table 5:** KMO test and Bartlett’s test of sphericity.

KMO sampling appropriateness measure		0.502
Bartlett’s test of sphericity	Approximate chi-square	295.004
	Degree of freedom	36
	Significance	< 0.001

#### Natural density and dry unit weight

This study selected nine performance indices of soil samples from 20 points: natural density, dry unit weight, moisture content, a 50 kPa shear strength, a 100 kPa shear strength, a 150 kPa shear strength, a 200 kPa shear strength, cohesive force, and internal friction angles, for PCA. Table 6 lists the total variances explained, from which it can be observed that the first three principal components can account for 78.896% of all variances. It suggests that the extracted three principal components, y1, y2, and y3, can represent 78.896% of the original nine performance indices. The extracted principal components for evaluating soil properties were affected by mining-induced subsidence to a certain degree.

**Table 6:** Total variance explained.

Component	Initial eigenvalue			Extract the sum of loads squared		
	Total	Percentage of variance	Accumulation %	Total	Percentage of variance	Accumulation %
1	3.236	35.956	35.956	3.236	35.956	35.956
2	2.576	28.621	64.577	2.576	28.621	64.577
3	1.289	14.319	78.896	1.289	14.319	78.896
4	0.743	8.254	87.150			
5	0.646	7.182	94.332			
6	0.451	5.011	99.343			
7	0.049	0.544	99.887			
8	0.010	0.113	100.000			
9	3.087E−06	3.430E−05	100.000			

#### Calculation of principal component (PC) coefficient

Using SPSS software, the component matrix was obtained, as listed in Table 7.

**Table 7:** Component matrix.

Index	Component		
	1	2	3
50 kPa shear strength	0.896	− 0.316	0.101
cohesive force *C*	0.792	− 0.566	0.015
150 kPa shear strength	0.767	0.502	0.273
200 kPa shear strength	0.666	0.610	0.374
100 kPa shear strength	0.639	0.192	− 0.063
Internal friction angle	− 0.231	0.928	0.176
Dry unit weight data	− 0.104	0.706	− 0.333
Natural moisture content	− 0.324	− 0.366	0.709
Natural density data	0.443	− 0.028	− 0.645

As shown in Table 7, for PC $y_{1}$, the absolution values of the coefficients for a 50 kPa shear strength ($x_{1}$), cohesive force C ($x_{2}$), a 150 kPa shear strength ($x_{3}$), a 200 kPa shear strength ($x_{4}$), and a 100 kPa shear strength ($x_{5}$) exceeded the values of the other indices, suggesting that PC y_2_ most comprehensively reflected the above five indices; for PC (y_2_), the absolution values of internal friction angle ($x_{6}$) and dry unit weight data ($x_{7}$) exceeded the values of the other indices, suggesting that PC y_2_ comprehensively reflected these two indices; for PC (y_3_), the absolution values of the coefficients of moisture content ($x_{8}$) and natural density ($x_{9}$) exceeded the values of the other indices, suggesting that PC y_3_ comprehensively reflected these two indices.

According to the coefficients of the above three PCs, the linear combinations of $y_{1}$ $y_{2}$ and $y_{3}$ can be written as:

11 $$y_{1} = 0.498x_{1} + 0.440x_{2} + 0.426x_{3} + 0.370x_{4} + 0.355x_{5} - 0.128x_{6} - 0.058x_{7} - 0.180x_{8} + 0.246x_{9}$$

12 $$y_{2} = - 0.197x_{1} - 0.353x_{2} + 0.313x_{3} + 0.380x_{4} + 0.120x_{5} + 0.578x_{6} + 0.440x_{7} - 0.228x_{8} - 0.017x_{9}$$

13 $$y_{3} = 0.089x_{1} + 0.014x_{2} + 0.241x_{3} + 0.329x_{4} - 0.055x_{5} + 0.155x_{6} - 0.293x_{7} + 0.624x_{8} - 0.568x_{9}$$

#### PC scores and comprehensive score

The comprehensive score can be calculated as follows:

14 $$y = 0.360y_{1} + 0.286y_{2} + 0.143y_{3}$$

Table 8 lists the scores of PCs calculated by principal component equations and the comprehensive scores calculated by using the ratio of variance contribution rates of each PC to the total variance contribution rates of two PCs as the weight. The following conclusions can be drawn from the calculated scores of various PCs and the comprehensive score. For the soil samples at depths of 60–80 cm and 20–40 cm in the moderate-subsidence area, the score of PC y_1_ was high, suggesting that the shear strength under a 50 kPa overburden pressure, cohesive force C, and shear strengths under 150, 200, and 100 kPa overburden pressures were most significantly affected after the occurrence of subsidence. For the soil samples at a depth of 0–10 cm in slight-subsidence and serious-subsidence areas, the score of PC y_2_ was high, suggesting that the internal friction angle and dry unit weight were most significantly affected after the occurrence of subsidence. For the soil samples at a depth of 40–60 cm in the moderate-subsidence area, at a depth of 20–40 cm in the serious-subsidence area, and a depth of 10–20 cm in the slight-subsidence area, the score of PC y_3_ was high, suggesting that moisture content and natural density were most significantly affected after the occurrence of subsidence.

**Table 8:** Scores of PCs and comprehensive scores.

Soil sample label	The score of PC *y1*	The score of PC *y*_2_	The score of PC *y3*	Comprehensive scores	Ranking
A1	2.28	2.42	0.28	1.55	1
A2	− 1.86	1.56	1.9	0.05	8
A3	− 0.85	0.1	− 0.33	− 0.33	13
A4	− 1.23	0.84	1.35	− 0.01	9
A5	0.36	− 2.3	− 0.03	− 0.53	16
B1	0.7	− 1.39	0.01	− 0.15	11
B2	1.68	1.05	− 0.1	0.89	4
B3	2.65	0.73	− 1.56	0.94	3
B4	− 1.84	0.88	− 0.5	− 0.48	15
B5	1.06	− 1.75	0.75	− 0.01	10
C1	− 0.26	− 0.55	− 0.83	− 0.37	14
C2	− 2.07	− 0.09	− 0.8	− 0.88	18
C3	− 3.04	− 0.94	− 0.64	− 1.45	20
C4	0.07	0.48	− 2.85	− 0.25	12
C5	4.36	− 0.93	1.5	1.52	2
D1	− 0.14	− 4.04	0.39	− 1.15	19
D2	− 0.3	1.74	0.22	0.42	6
D3	− 1.12	1.1	1.98	0.19	7
D4	− 1.08	− 0.84	− 0.47	− 0.7	17
D5	0.64	1.94	− 0.29	0.74	5

Regarding comprehensive scores, the soil samples at a depth of 0–10 cm in the slight-subsidence area and at a depth of 60–80 cm in the moderate-subsidence area were most significantly affected by mining-induced subsidence, with comprehensive scores of over 1.5. After subsidence, these two types of soil samples underwent the most intense structural changes, and soil density, basic physical properties, and shear strength indices showed apparent differences from the original values. This is because, for unsubsidence areas and mild subsidence areas, the impact of subsidence on soil vertically shows a gradual decrease with the increase of soil depth, while for moderate and severe subsidence areas, the impact of subsidence on soil decreases first and then increases with depth, that is, the impact is the least in the middle layer and the greater in the surface layer and deep layer. It can be seen that mechanical properties are more or more affected by coal mining collapse than physical and chemical properties, mainly because coal mining collapse will destroy the structure of soil and lead to changes in soil mechanical properties. Specifically, coal mining collapse will increase soil porosity and decrease soil density, thus reducing soil shear strength and cohesion.

## Discussion

Significant surface subsidence induced by coal mining changes surface soil’s physical and mechanical properties. It destroys the original ecological environment, seriously restricting the sustainable development of local society and the economy. This study focused on surface soil under the influence of mining-induced subsidence, which is significant and can provide a theoretical foundation for ecological remediation in mines. In this study, the conditions before and after mining-induced subsidence and under different subsidence degrees in the temporal dimension and soil samples at different depths in the spatial dimension were collected; meanwhile, basic physical indices and shear strength indices were selected. Therefore, the present test results can reveal and explain the influences of mining-induced subsidence on soil’s basic indices and conclude the variation rules of physical and mechanical indices of surface soil with different subsidence degrees and at different depths. Moreover, the distribution characteristics of various indices in horizontal and vertical space were investigated in depth. Using PCA, PCs were extracted from various indices. The comprehensive scores were calculated to examine the degrees of influence of mining-induced subsidence on various indices and explore the indices significantly affected by mining-induced subsidence, the indices at different sampling points, and the sampling points under significant influence.

The soil dry unit weight was seriously affected by mining-induced subsidence and dropped to a certain degree. The soil samples in the shallow layer were under great disturbance, while the soil samples in the middle were slightly affected since they were flattened on the two sides despite fluctuations. Zhou et al.^39–42^ focused on the effects of mining-induced subsidence on aeolian soil properties in Erdos and found that soil bulk density dropped gradually with sampling depth. This may be because the soil samples in their study were collected at a depth of 0–30 cm, while this study focused on the soil samples collected at a depth of 0–80 cm. Under mining-induced subsidence, surface soil was subjected to significant influence, while deep soil was slightly affected^43^; however, soil in the bottommost layer was enhanced to a certain degree.

Regarding the soil moisture content, the moisture content of soil at a depth of 20–40 cm significantly dropped after the occurrence of subsidence and increased again with increasing subsidence degree; at a depth of 10–20 cm, the moisture content in the nonsubsidence area was highest, which dropped after subsidence. This is consistent with the research results by Cui et al. The soil surface settles to varying degrees after coal mining, thereby imposing disturbance and damage to the surface soil. Accordingly, the soil structure was destroyed, and the migration rules of molecules in the soil were affected^44^. At the beginning of coal mining, many collapse fractures were produced on the surface in the subsidence area, which showed different forms, sizes, widths, and strikes. Because of the fractures, the contact area between the soil and the external environment increased, and the evaporation capacity increased, resulting in severe evaporation. This can also account for the variation in moisture content under mining-induced subsidence.

In previous studies, many scholars selected basic physical indices and some indices related to the plant root system and biocenosis and rarely selected mechanical indices. This study selected shear strength characteristics for indepth analysis, which can avoid the oneness in index selection and enhance the systematicness of soil property analysis. The present results revealed that slight and moderate subsidence could harden the soil structure in shallow and deep layers, leading to close contact among soil particles, increased internal friction, and enhanced shear strength; however, soil strength weakened under severe subsidence.

Considering personnel safety and previous classification standards, the region with a subsidence height of over 40 cm was defined as having a severe subsidence degree. The research region shows a significant span in subsidence height, and accordingly, the variation rules of basic properties in this study have certain limitations. Mining-induced subsidence is a complex process with a redistribution of stress. Different stresses impose different disturbances on basic soil properties. Underground stress distribution patterns should be combined to analyze the variation mechanism of indices. Accordingly, long-term monitoring of topographical changes in subsidence regions, long-term variation features of soil properties, and scientific identification of subsidence processes will be the focus of future research.

## Conclusions

This study focused on the mining-induced subsidence region in the No. 4 Mine, Yili, and investigated the variation characteristics of basic soil properties with subsidence degree and sampling depth.

1. For soil samples in the shallow layer, subsidence reduced the natural density and dry unit weight, with a maximum reduction of 7%. The moisture content increased with subsidence degree, with an overall increase of 4%. As the depth increased, the effects became less pronounced. At a large depth, both the natural density and dry unit weight increased by 0.07–0.14 g/cm^3^, while the moisture content was slightly affected by subsidence.
2. Under slight and moderate subsidence, the soil structure was hardened, accompanied by tighter contact among soil particles. Accordingly, the internal friction angle and soil shear strength were enhanced by 12.5–25%; however, under severe sub-sidence, the soil strength weakened, with a maximum reduction of 30.7%.
3. Among the selected nine indices in this study, shear strength under 50 K over-burden pressure and cohesive force in terms of shear property were subjected to the most significant influence, with the most apparent changes. In terms of physical prop-erty, dry unit weight was most significantly affected by mining-induced subsidence. Comparing physical and shear indices, the latter was more seriously affected by the mining-induced collapse. Among the 20 sampling depths with different subsidence degrees and sampling depths, the soil samples at 0–10 cm in the slight-subsidence area and 60–80 cm in the moderate-subsidence area were more significantly affected by the mining-induced collapse, with comprehensive scores of PCs of over 1.5.
4. This study revealed why mining-induced subsidence affected soil’s basic properties, concluded the variation rules of surface soil’s physical and mechanical indices with subsidence degree and sampling depth, and reasonably evaluated the quantitative effect on soil’s physical and mechanical indices. This research can contribute to gaining in-depth knowledge of the disturbance mechanism of mining-induced subsidence on the comprehensive properties of soil and provide theoretical support for establishing predictive models of key soil indices in different stress regions and at different recovery phases. This study is considered instrumental in formulating a reasonable development strategy and supporting ecological protection measures for protecting the ecological environment of coal mining areas in Xinjiang, China.

## Data Availability

The datasets used and/or analysed during the current study available from the corresponding author on reasonable request.
